# Fat, Sugar or Gut Microbiota in Reducing Cardiometabolic Risk: Does Diet Type Really Matter?

**DOI:** 10.3390/nu13020639

**Published:** 2021-02-16

**Authors:** Katarzyna Nabrdalik, Katarzyna Krzyżak, Weronika Hajzler, Karolina Drożdż, Hanna Kwiendacz, Janusz Gumprecht, Gregory Y. H. Lip

**Affiliations:** 1 Liverpool Centre for Cardiovascular Science, University of Liverpool and Liverpool Heart & Chest Hospital, Liverpool L14 3PE, UK; Gregory.Lip@liverpool.ac.uk; 2 Department of Internal Medicine, Diabetology and Nephrology, Faculty of Medical Sciences in Zabrze, Medical University of Silesia, 40-055 Katowice, Poland; karolina.drozdz92@gmail.com (K.D.); hkwiendacz@sum.edu.pl (H.K.); jgumprecht@sum.edu.pl (J.G.); 3 Students’ Scientific Association by the Department of Internal Medicine, Diabetology and Nephrology, Faculty of Medical Sciences in Zabrze, Medical University of Silesia, 40-055 Katowice, Poland; kkrzyzak18b@gmail.com (K.K.); hajzlerweronika@gmail.com (W.H.); 4 Aalborg Thrombosis Research Unit, Department of Clinical Medicine, Aalborg University, 9100 Aalborg, Denmark

**Keywords:** carbohydrates, diabetes mellitus, diet, fat, microbiota

## Abstract

The incidence of cardiometabolic diseases, such as obesity, diabetes, and cardiovascular diseases, is constantly rising. Successful lifestyle changes may limit their incidence, which is why researchers focus on the role of nutrition in this context. The outcomes of studies carried out in past decades have influenced dietary guidelines, which primarily recommend reducing saturated fat as a therapeutic approach for cardiovascular disease prevention, while limiting the role of sugar due to its harmful effects. On the other hand, a low-carbohydrate diet (LCD) as a method of treatment remains controversial. A number of studies on the effect of LCDs on patients with type 2 diabetes mellitus proved that it is a safe and effective method of dietary management. As for the risk of cardiovascular diseases, the source of carbohydrates and fats corresponds with the mortality rate and protective effect of plant-derived components. Additionally, some recent studies have focused on the gut microbiota in relation to cardiometabolic diseases and diet as one of the leading factors affecting microbiota composition. Unfortunately, there is still no precise answer to the question of which a single nutrient plays the most important role in reducing cardiometabolic risk, and this review article presents the current state of the knowledge in this field.

## 1. Introduction

The incidence of cardiometabolic diseases is increasing, with a worldwide epidemic of obesity, diabetes, and atherosclerotic cardiovascular diseases. Since 1975, the prevalence of obesity has almost tripled and, in 2016, the number of obese patients exceeded 650 million [1]. In the United States, it has been predicted that, by 2030, almost one in two adults will be obese, and the prevalence will be higher than 50% in most states and not below 25% in any state [2]. This situation is similar for diabetes mellitus, where, in 2019, the number of patients aged between 20 and 79 years was estimated to be 463 million worldwide [3]. Obesity and diabetes are both strong risk factors for cardiovascular diseases. It is estimated that more than 700,000 deaths per year in the United States alone are caused by cardiometabolic diseases and approximately 50% of them are related to diet [4]. World Health Organization (WHO) statistics state that, worldwide, 17 million people die of cardiovascular diseases (CVD) annually [5]. Diet type is one of the interventions, besides physical activity, influencing cardiometabolic health. Since the beginning of the 20th century, there has been an ongoing debate on which diet type is favorable in relation to cardiometabolic health, which is, whether this should be a low-fat diet (LFD) or a low-carbohydrate diet (LCD). Recently, a third aspect to this debate has been introduced, namely the gut microbiota, which are involved in the cardiometabolic health of the host through diet type, which complicates the present knowledge gap that is related to the influence of diet on metabolic health [6].

Hence, there is still no precise answer to the question of which the type of nutrient plays the most important role in the reduction in cardiometabolic risk and this review article presents the current state of the knowledge in this field.

## 2. Epidemiological Insights

In the middle of the 20th century, coronary heart disease (CHD) was recognized as an epidemic [7]. This started a series of studies on the causes of this disease. In 1908, when a link was established between a high cholesterol intake and the faster development of atherosclerosis in rabbits [8], it was realized that the CVD in humans could be related to nutrition. A prophylactic change in diet, especially in combination with physical activity, which would reduce the risk of developing CVD by even a small percentage, may significantly affect the number of people who will develop CVD and, thus, reduce the cost of patients’ care [9].

In the mid-1950s, the first studies on the influence of the presence of fat in the diet on the risk of developing CVD began [10]. In 1958, the Seven Countries Study (SCS) was launched, the aim of which was to collect data and deepen the knowledge regarding the variables that are likely to be relevant for the CHD epidemiology [11]. In the SCS, 16 culturally diverse cohorts (southern, central and northern Europe, Japan, Italy, and the United States) were selected, representing a wide range of differences in dietary fat and carbohydrate intake. Keys et al. noted that the differences between populations in the incidence of CHD are related to lifestyle, especially the type of diet, particularly its fat content [12]. The intake of large amounts of this macroelement resulted in an increase in the total plasma cholesterol concentration, one of the first CVD risk factors identified.

High serum cholesterol concentration is correlated with a high risk of mortality from CHD [13]. The recommendations for the reduction in saturated dietary fats to reduce CVD risk appeared in the American Heart Association (AHA) guidelines as early as in 1961 [14]. In 1970, the preliminary results of the SCS were published, showing a lower incidence of CVD in inhabitants of the Mediterranean coast, Greece, southern Italy, and former Yugoslavia [15]. The diet from these regions was referred to as the Mediterranean Diet (MED), and it differed from the American and North European diets, in that it contained much less meat and dairy products and more fruit, vegetables, and whole grain products [15,16,17]. The main sources of fat were olive oil and nuts [17]. This diet seemed to be a factor determining the large difference in CVD prevalence between the populations of the Mediterranean coast and Western cohorts in the SCS [15].

However, this hypothesis was not matched by epidemiological data that were collected from the French population. It was observed that, despite the high level of saturated fat intake, CVD-related mortality was low [18,19]. This phenomenon was called the French Paradox. Studies have shown that the consumption of wine and its phenolic compounds in moderate quantities, as in the French culture, can have a cardioprotective effect [20]. A direct effect on the development of atherosclerosis causes this, increasing the level of high-density lipoprotein cholesterol (HDL-C), as well as by the hemostatic mechanism, which prevents platelet aggregation [19,21]. It is now known that the phenolic compounds that are derived from wine will also reduce low-density lipoprotein cholesterol (LDL-C) oxidation, oxidative stress, cause an increase in NO (nitrogen oxide) release, and, in this way, improve the endothelial function [22,23]. The effects of these compounds may cause the so-called French Paradox.

At the end of the 1950s, a debate emerged as to whether fat or sugar would be a worse diet macronutrient in relation to heart diseases [24]. Keys (via the SCS) claimed that dietary fat was to blame for the increasing number of heart diseases [25]. Meanwhile, a British nutritionist, John Yudkin, blamed carbohydrates—primarily refined sugars—given that sugar consumption rose in parallel with the increase in heart diseases when people broadly consumed meals that were low in fat [26,27].

For decades, starting from 1980, dietary guidelines recommended lowering the total fat and saturated fatty acids (SFA) [28], and these were updated in 1990 in order to recommend LFDs, specifically consisting of ≤30% of total fat and ≤10% SFA of the total daily energy [29]. At the same time, as fat decreased in the American diet [30], there was a rise in the consumption of refined grains [31] and an increase in the prevalence of type 2 diabetes mellitus (T2DM) and CVD [32,33]. Because obesity is a well-recognized risk factor for T2DM, there has been increasing interest in LCD for weight loss, especially since the 1970s, when “Dr. Atkins’ New Diet Revolution” became a worldwide phenomenon [34]. In fact, the history of LCD began in 1797, when John Rollo described two cases of soldiers with T2DM that was treated with carbohydrate restriction [35,36] and, later on, in 1869, when William Banting, in his open letter, proposed LCD as a successful method to lose weight. Banting himself lost 46 pounds (approximately 21 kg), when his acquaintance, Claude Bernard, prescribed him an LCD regime [37,38]. Over the years, LCD has had supporters and opponents. This diet permanently became the subject of research into dietary approaches not only due to its ability to reduce body weight, but also because of its role in the prevention and treatment of many diseases. LCD remains controversial, but there has recently been increased interest in this type of diet [34,39,40,41,42,43,44]. In fact, the exact amount of carbohydrate to be eaten daily for optimal health is unknown [45], although the recommended daily carbohydrate intake is approximately 45% of total calorie intake [46]. According to Feinman et al., the definition of LCD is consuming less than 130 g of carbohydrates per day and less than 26% of energy from carbohydrates [47]. Currently, the WHO and other worldwide authorities emphasize how important the type of carbohydrates consumed is: the preferred ones are unrefined carbohydrates rich in fiber, vitamins, and minerals with simple sugars being limited to a maximum of 10% of total calories per day [48]. An extreme type of LCD is a very low-carbohydrate diet (VLCD), which contains less than 20 to 50 g of carbohydrates and below 10% of energy from carbohydrates [47]. A special type of this kind of diet is the ketogenic diet, which combines a very low carbohydrate, high fat, and moderate protein consumption [49]. In 2019, The American College of Cardiology (ACC)/AHA guidelines on the primary prevention of CVD introduced guidance on diet counseling [50]. Within these guidelines, there was emphasis placed on a whole foods approach, rather than focusing on a single nutrient, encouraging a higher intake of fresh vegetables and fruits and limiting the consumption of processed meats and sugary beverages to reduce the atherosclerotic cardiovascular disease (ASCVD) risk [50].

**Figure 1:**
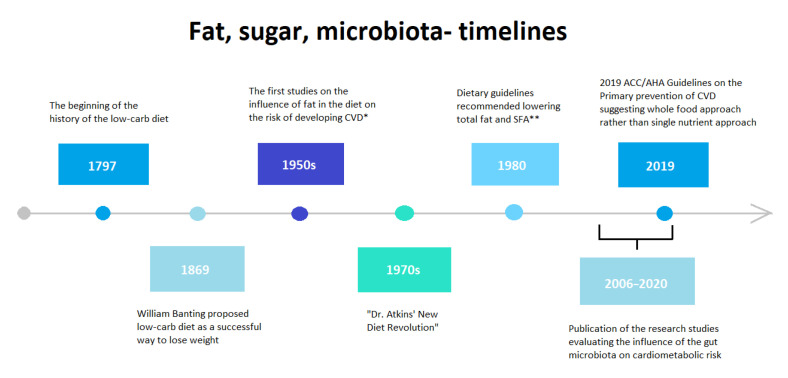
Fat, sugar and microbiota timelines. * CVD: cardiovascular disease; ** SFA: Saturated fatty acids.

More recent years have highlighted another link to diet and cardiometabolic disease, the gut microbiota, which are involved in the metabolic control of the host [51]. Dysbiotic gut microbiota are thought to be related to cardiometabolic diseases, such as obesity [52], T2DM [53], and CVD [54,55,56], which is why gut modulation strategies, like diet intervention, may provide some possibility for reducing cardiometabolic risk through correcting the microbial gut imbalance. Figure 1 summarizes the timelines related to fat, sugar, and microbiota studies.

## 3. Low-Fat Diet and Obesity

The general fact is that it is impossible to lose weight without a negative energy balance [57] and, in addition to the daily energy reduction, macronutrient composition has been an important issue that is examined in various studies for many years. LFDs for weight loss were recommended due to the conviction that energy from fat is less satiating when compared to carbohydrates [58], as carbohydrate is more thermogenic than fat and [59] high fat intake may cause intestinal dysbiosis with a detrimental impact on metabolic variables [60].

Data from a meta-analysis of studies assessing LFDs and LCDs’ influence on weight loss, when comparing results from 48 randomized trials (total 7286 participants, median age of 45.7 years, median BMI of 33.7), showed that both LFD and LCD were associated with similar body weight loss in 12 months, and the differences between them were minimal (LCD—7.25 kg (95% CI, 5.33 to 9.25 kg) and LFD—7.27 kg (95% CI, 5.26 to 9.34 kg) [61]. LCD and LFD both reduced body weight by 8 kg on average in a 6-month observation compared to no diet. Approximately 1 to 2 kg of this effect was lost during the 12-month observation. This confirms the thesis that most calorie-lowering diets lead to clinically significant weight loss as long as the diet is maintained. Indeed, it is important to choose a diet that will be best tolerated by the patient, as the time spent following the diet is more important than the content of individual macronutrients [61]. Similarly, the Diet Intervention Examining The Factors Interacting with Treatment Success (DIETFITS) trial, assessing the effect of LFDs vs. LCDs on 12-month weight loss, also revealed that there is no significant difference in the weight loss between those two types of diets [62]. Meckling et al. observed the same outcome [63] The results are largely in line with the recommendations that were published by the Joint Guidelines from the AHA, ACC, and the Obesity Society [64], which stress the importance of following healthy eating patterns, which could be DASH (Dietary Approaches to Stop Hypertension) diet or the Healthy Mediterranean-Style Eating Pattern, instead of simply identifying that one diet is superior to the others. However, studies assessing LFD and LCD are still performed, and Chawla et al. recently proved, in their meta-analysis, that LFD had a lesser effect on weight loss when compared to LCD [65]. Table 1 summarizes the trials focusing on LFD in obesity.

## 4. Low-Fat Diet in Type 2 Diabetes Mellitus

An acceptable consumption of total fat for all adults is said to be 20–35% of total daily calorie intake [45]. There is a need to look at the type and quality of fat rather than quantity, because it may influence CVD [66,67,68] and synthetic sources of trans fats need to be avoided [28,69,70]. Additionally, a systematic review and meta-analysis indicate that lowering the total fat intake does not necessarily improve glycaemia and CVD risk [71,72,73], and the positives from LFD are mostly related to weight loss [69,74]. Lately, the evidence seems to indicate that the major aspect for CVD prevention is the quality of fat consumed, rather than the total amount of fat intake [66]. In relation to the quality of fat, it is important to look at MUFA (monounsaturated fatty acids), PUFA (polyunsaturated fatty acids), and SFA (saturated fatty acids) [75]. Although their division into three major groups is helpful in determining structural affiliation, it may lead to oversimplified conclusions regarding the effect of fat type on cardiovascular risk [76]. PUFAs can be divided into n-6 and n-3 PUFAs, derived from linoleic acid (LA) and a-linolenic acid (ALA). These acids are not synthesized in the human body; therefore, they must be consumed [75]. Replacing SFAs with PUFAs has so far shown the best effect on lipid profile, but the research results vary, depending on which lipids are studied [75,76]. In addition to its inhibitory effect on atherosclerosis, MUFA may also have a role in lowering blood glucose concentration, which could be important for patients with diabetes [70,76]. LFD appears to have a significantly smaller effect on T2DM control than LCD [77,78], but, as was proved in other studies, the differences in the results are statistically insignificant [79,80,81]. Table 2 summarizes the studies assessing LFD and high-quality fat in patients with T2DM.

## 5. Low-Fat Diet and Cardiovascular Risk

The caloric demand is covered by three main macronutrients: fats, carbohydrates, and proteins. A reduction in the intake of one component leads to an increase in the intake of another to maintain the energy balance.

The impact of reduced saturated fat intake on the development of CVD is highly dependent on the ingredients that replace it [20,82,83,84,85]. In the studies where energy from saturated fats was mostly replaced by carbohydrates, there was no significant reduction in the CVD incidence observed [9,86,87]. Favorable correlations occurred where saturated fats were replaced by unsaturated fats, especially PUFA, which suggests that the final results of the study may have been influenced by an increased consumption of polyunsaturated fats [88,89].

The most predictive measure for CHD is not total plasma cholesterol concentration, but the ratio of total cholesterol to HDL-C [90]. Saturated fats increase the concentration of both HDL-C and LDL-C, which has minimal effect on the ratio of total cholesterol to HDL-C. However, there is evidence that the replacement of saturated fats by PUFA, such as omega-3 ALA or docosahexaenoic acid (DHA), leads to a reduction in atherosclerosis development and, thus, CHD [91,92,93]. PUFAs mainly act by improving the lipid profile by lowering total cholesterol levels, triglycerides and LDL-C. Moreover, they have a positive effect on atherosclerotic plaque stability, platelet aggregation, concentration of proinflammatory cytokines, and immune cells [94]. Furthermore, omega-3 PUFAs have a beneficial effect on endothelial progenitor cell biology [95]. Omega-3 fatty acid supplementation is currently used to reduce the risk of cardiovascular events [94]. In the treatment and prophylaxis of CVD, the most important direction is the introduction of a healthy, balanced diet, while taking the controlled content of fatty acid into account [92].

Since the pioneering SCS, many randomized clinical trials (RCT) and meta-analyses of observational and RCT studies have been conducted. The results have led to heterogeneous conclusions regarding the relationship between saturated fat intake and CVD development risk [89,96,97,98,99].

The largest intervention study PREDIMED (Prevención con Dieta Mediterránea), on the use of the MED, showed that, among the participants (patients without CVD at the beginning of the study, but with a high risk of developing these conditions), a lower incidence of cardiovascular events during the five-year observation period was noticed in people who were on a diet with olive oil or nuts than in people on LFD [100]. In 2017, concerns were raised regarding the PREDIMED study, especially irregularities in the randomization procedures; therefore, in June 2018, the basic report was retracted [100] and republished [101] in a corrected form, which took any deviations in the conduct of the study into account, although the conclusions remained the same. Table 3 summarizes the associations between low-fat and high-quality fat diets and cardiovascular risk.

## 6. Low-Carbohydrate Diet in Obesity

Carbohydrate restriction causes an increase in glycogenolysis, gluconeogenesis, and fat oxidation to maintain proper blood glucose concentration [102,103]. One hypothesis as to why LCD is effective in weight loss is that the aforementioned processes require more energy expenditure; however, this hypothesis requires further confirmation [104,105]. Carbohydrate limitations, e.g., to 50 g per day, cause ketogenesis—an increased production of ketone bodies, such as acetoacetate, β-hydroxybutyric acid, and acetone—as an alternative source of energy [106]. Ketogenesis suppresses the appetite, which is one of the ways in which weight can be lost on a ketogenic diet [104,107].

In their meta-analysis, Gibson et al. found that people on ketogenic diets were less hungry and had a reduced desire to eat. The ketogenic diet protected them from an increased appetite, despite weight loss [107]. A higher consumption of protein may also have a satiating effect, because of the elevated level of satiety hormones [108]. LCD results in a reduction in circulating insulin concentration, which promotes the transfer of triacylglycerol into free fatty acids and glycerol [109,110]. Free fatty acids and glycerol are used by muscles, and this results in a reduction in fat tissue and weight loss [109,110]. Another theory as to why the LCD is effective in weight loss is that using this diet causes a reduction in the overall calorie intake in practical terms [111]. The regime of the LCD means that food choices are limited, which may lead to weight loss [111].

The results of studies concerned with the effects of LCDs on body weight vary, but mainly indicate positive results, especially in the short term, and demonstrate better effects than other diets [112,113,114,115,116,117,118,119,120]; however, Foster et al., in their study, which lasted two years, did not find any difference between the compared diets [121]. LCD and VLCD may be advantageous in relation to appetite, triglyceride, and medication use in T2DM, with no clear evidence as to the advantages in terms of cardiometabolic risk [122]. Moreover, the ketogenic diet resulted in better long-term body weight management with greater reductions in body weight than LFD [117]. Table 4 summarizes the results of these studies.

## 7. Low-Carbohydrate Diet in Type 2 Diabetes Mellitus

The European Society of Cardiology (ESC), European Association for the Study of Diabetes (EASD), and American Diabetes Association (ADA) recommendations [45] state that there is no single ideal dietary distribution of calories among carbohydrates, fats, and proteins for T2DM patients, emphasizing the role of maintaining normal body weight in this condition [45,123].

The fundamental issue of the implementation of LCD in the lifestyles of T2DM patients is maintaining this type of diet, especially in individuals that are treated with insulin, because of the potentially increased risk of ketosis and hypoglycemia. Before the miraculous discovery of insulin in the 1920s, the restriction of carbohydrates appeared to be one of the ways in which patients with T2DM could be kept in better condition [124]. LCD was used by Joslin in 1893 and Allen in 1914 in patients suffering from T2DM with varying results, but they did have therapeutic success in some cases [125,126,127]. The improvement of T2DM control with a dietary approach is essential, and one of the possible therapeutic agents seems to be an LCD.

There are many studies, meta-analyses, and systematic reviews regarding LCDs’ efficiency in T2DM patients [73,77,80,128,129,130,131,132,133]. A meta-analysis carried out by Turton et al. concluded that LCD intervention for T2DM management is safe and effective [128], and one prospective Japanese study showed that LCDs were associated with decreased risk of T2DM in women [129]. A meta-analysis cited in the ECS/EASD guidelines from 2019 [123] indicates that the glucose-lowering effect of low- and high-carbohydrate diets is similar after 1 year or more and there is no significant effect on the weight or LDL-C levels [134]. The main benefit of LCDs seems to be better glycemic control in T2DM, especially their effect in terms of lowering HbA_1c_ [77,130,131,132]; however, this benefit seems to be short term, and there is controversy that is related to the Japanese population. LCD has a positive role in lowering the dosages of insulin and fasting blood glucose [80]. The ketogenic diet also seems to substantially reduce the glycemic response that results from dietary carbohydrates, as well as improving the underlying insulin resistance [133]. Table 5 summarizes these studies.

## 8. Low-Carbohydrate Diet and Cardiovascular Risk

There is controversy regarding restrictions related to carbohydrates, as attempts to prevent cardiovascular risk began in the middle of the 21st century and, since that time, numerous studies have been performed with mixed results, which means that the role of LCD in patients with T2DM is unclear (EASD), as mentioned above [135,136,137,138,139,140]. After 20 years of follow up, Halton et al. observed that diets lower in carbohydrates and higher in protein and fat were not associated with an increased risk of CHD in women and, when the source of the protein or fat was vegetables, the risk of CHD was moderately reduced [135]. One prospective cohort study indicated that LCD was associated with an increased risk of atrial fibrillation, regardless of the type of protein or fat used to replace the carbohydrates [136]. High- and low-carbohydrate diets were both associated with an increased mortality, with minimal risk being observed at the daily carbohydrate intake of about 50–55% [137]. LCDs with mainly animal-derived protein and fat sources were associated with higher mortality, but LCDs consisting of plant-derived protein and fat sources were associated with lower mortality [137]. LCDs were associated with a significantly higher risk of all-cause mortality and they were not significantly associated with a risk of cardiovascular mortality and incidence [138]. Low-carbohydrate, high-protein diets, without consideration of the source of protein, were also associated with increased cardiovascular risk in Swedish women [140]. An interesting perspective is the impact of LCDs on cardiovascular risk factors, such as dyslipidemia, hypertension, obesity, postprandial hypoglycemia, and endothelial dysfunction [111,112,117,118,141,142]. A randomized trial comparing VLCD and calorie-restricted LFD in women with obesity indicated that VLCD was more efficient in short-term weight loss [111]. The mean levels of blood pressure, lipids, fasting blood glucose, and insulin were within the normal ranges in both groups [111]. LCD was associated with a significantly lower predicted risk of atherosclerotic cardiovascular disease events than LFDs in overweight and obese adults [112]. VLCD decreased body weight, triglyceride concentration, and diastolic blood pressure, while increasing the concentration of HDL-C and LDL-C [117]. A systematic review demonstrated that LCDs were more effective at six months than LFDs in reducing the weight and cardiovascular disease risk [118]. Another study proved that, in young adults of normal weight on LCDs for three weeks, LDL-C increased by 44% vs. the control group [141]. Only one week of an LCD leads to a relative impairment in glucose homeostasis in healthy young adults [142]. The authors stated that this process may predispose the endothelium to hyperglycemia-induced damage, but there is a need for further studies on young, healthy men [142]. The most recent meta-analysis that is related to the effects of LCD on CVD risk factors confirmed that this type of diet has a beneficial effect on cardiovascular risk, but long-term studies are needed in order to confirm this [143]. As we know, T2DM is an important cardiovascular risk factor itself and studies including T2DM patients also analyzed the other cardiovascular risk factors that are mentioned above [144,145,146,147]. LCD intervention in patients with T2DM had a positive effect on reducing triglyceride concentration and increasing HDL-C concentrations, without a significant effect on long-term weight loss [144]. Another study indicated that an LCD approach in patients with T2DM for an average of two years caused a significant reduction in blood pressure, weight, and an improvement in lipid profiles [146]. Table 6 summarizes the results of the studies related to LCD and CVD risk.

## 9. Fat and Sugar—New Insights

The debate regarding which single nutrient is the most important for reducing in cardiometabolic risk is still ongoing, because, to date, the results of studies on this topic have many limitations. Unlike previous studies [138,148], one prospective population-based study [149] investigated the associations of not only macronutrients, but also their components, with all-cause mortality and CVD. In that study, by the UK Biobank, there were 502,536 participants recruited (aged 37–73 years) in 2007–2010, of whom 211,023 completed at least one dietary questionnaire and 195,658 were eligible for the study. There was a mean follow-up period for a mortality of 10.6 years. The main finding was that carbohydrates (sugar, starch, fiber) and proteins were non-linearly associated with all-cause mortality. Similarly, a non-linear association was found for fiber, PUFA, and protein with incidences of CVD. On the other hand, a linear association was observed between the intake of SFA, MUFA, and PUFA for all-cause mortality and for total carbohydrate and total fat with incident CVD. There was a lower risk of all-cause mortality and incident CVD among patients whose current intake of starch, MUFA, and protein was low and had sugar replaced with starch, MUFA, or protein. Moreover, replacing SFA with MUFA or protein lowered the risk of the total mortality and CVD incidence. An important observation coming from this study is that a divergent association of sugar and starch with all-cause mortality can be found, and one should look not only at the amount, but also at the components of carbohydrates. However, it must be also emphasized that the current intake of a macronutrient should be considered, since the all-cause mortality was lowered only among those patients who had sugar replaced with starch at the time when their current intake of starch was low. As such, it cannot be generalized that replacing sugar with starch in patients consuming higher amounts of starch will derive the same result. There are also studies analyzing not only carbohydrate compounds, but also carbohydrate quality, such as glycemic index (GI) or glycemic load (GL), which rank carbohydrates according to the ability to increase blood glucose concentration. The results from a recently published pan-European cohort study indicate that high GL or GI diets, which lead to a high glucose response, are associated with higher CVD risk [150]. In addition to the amount and quality of macronutrients, their production practices may also influence CVD, which has been recently proven in a population-based cohort study [151]. In this study, a large amount of ultra-processed foods in the diet was associated with higher risks of cardiovascular, coronary heart, and cerebrovascular diseases [151].

## 10. Low-Fat, Low-Carbohydrate Diets in Relation to Microbiota in Cardiometabolic Risk

In recent years, it has become apparent that there is a relationship between diet, gut microbiota, and metabolic health, including obesity and cardiovascular diseases. The gut microbiota composition differs between obese and lean subjects; for instance, in obese subjects, the Firmicutes to Bacteroides ratio is elevated, and a higher proportion of Actinobacteria as well as reduced bacterial diversity is observed [152]. In relation to T2DM, a lower abundance of fiber-degrading bacteria has been found [153], as well as a reduction in Firmicutes phyla and an increase in Bacteroides to Firmicutes and Bacteroides to Pravotella ratios [154]. Both of the dietary approaches, namely LFD or LCD, may (but also may not) lead to weight loss, and there is substantial variability in the results in this regard. One explanation for this may be the difference in gut microbiota. Food intake can be reduced due to the influence of gut hormones that are stimulated by products of microbial fermentation, such as butyrate and propionate [155]. Dietary adherence has become a major limitation for sustained weight loss, which leads to a reduction in CVD risk [156].

One recent focus has been on the gut microbiota’s involvement in sustained weight loss potential. Grembi et al., examining a cohort of obese adults enrolled in the DIETFITS trial, proved that structured differences in gut microbiota may explain a portion of the variability in weight loss success [157]. In that study, when determining whether gut microbiota could predispose participants to successful 12-month weight loss following LCDs or LFDs, the authors concluded that long-term weight loss is correlated with gut microbiota variability in a diet-dependent manner. Patients who were on LFDs and had higher pre-diet microbiota plasticity had more sustained weight loss, whereas patients who were on an LCD and had higher microbiota variability over 10 weeks of dietary treatment had increased 12-month weight loss. To the best of our knowledge, there is only one study that directly relates the effect of dietary fat on gut microbiota and addresses their relationship with cardiometabolic diseases. In this study, Wan et al. [158] showed that higher fat consumption was associated with unfavorable changes in the gut microbiota, as well as fecal metabolomics and plasma proinflammatory factors.

High-fat and high-sugar Western diets negatively impact on human metabolic health through alterations in the gut microbiota. Microbiota-accessible carbohydrates, which are found in dietary fiber, shaping the microbial ecosystem, are reduced in Western diets [159]. In addition, the microbiota can affect cholesterol balance and, through this mechanism, CHD development [160]. The first studies that proved the potential link between the gut microbiome and CVD analyzed trimethylamine N-oxide (TMAO), which is a metabolite arising from the ingestion of dietary nutrients that are abundant in a Western diet, namely lecithin, choline, and carnitine [161]. In turn, there is an association between TMAO concentration and increased cardiovascular risk and mortality, as observed in large-scale clinical cohorts [162].

It is also worth noting that food production practices and additives (e.g., emulsifiers and non-caloric artificial sweeteners) influence human health, including the gut microbiota [163,164].

## 11. Conclusions

The question of which type of diet—LCD or LFD—is better for cardiometabolic health remains unanswered. LCDs and LFDs can both be successful in relation to weight loss (which, in turn, indirectly improves cardiovascular risk), but the differences may be dependent on the host microbiome. Food production practices, fiber content, carbohydrate sources, and fatty acid quality may play a significant role in cardiovascular risk management. Identifying features of the gut microbiota that can predict adherence to the specific diet type and may help to personalize dietary interventions will be necessary.

## Figures and Tables

**Table 1:** Low-fat diet and obesity.

Authors (Year) Study Type	Studies/Participants (*N*) Average Duration of Follow-Up	Population	Diet Type Compared	Findings (Weight Loss/Hunger)
Meckling et al. (2004) RCT [63]	1/31 10 weeks	Overweight and obese adult men and women	LFD vs. LCD	No significant differences in body weight between the studied groups of patients
Johnston et al. (2014) [61] Meta-analysis, RCT	48/7286 6 and 12 months	Overweight and obese (BMI ≥ 25 kg/m^2^) adults without co-morbidities.	LCD vs. LFD vs. no special diet	Weight loss observed on LCD was: 8.73 kg (95% CI: 7.27 to 10.20 kg) after 6 months of follow-up and 7.25 kg (95% CI: 5.33 to 9.25 kg) after 12 months. Weight loss on LFD was 7.99 kg (95% CI, 6.01 to 9.92 kg) after 6 months of follow-up and 7.27 kg (95% CI, 5.26 to 9.34 kg) after 12 months. There were no significant differences between diets.
Gardner CD et al. (2018) [62] RCT, DIETFITS	1/609 1 year	Adults without diabetes with a BMI 28–40	LFD vs. LCD	There was no significant difference in weight loss.
Chawla et al. (2020) [65] Systematic review and meta-analysis of RCT	38/6499 6 and 12 months	Healthy adult, BMI 22 and 43.6 kg/m2	LCD vs. LFD	LCD at 6–12 months was favored for average weight change—polled analyses—mean difference −1.30 kg; 95% CI −2.02 to −0.57

Abbreviations: BMI—Body Mass Index; T2DM—type 2 diabetes mellitus; LCD—low-carbohydrate diet; LFD—low-fat diet. RCT-randomized controlled trial; DIETFITS - Diet Intervention Examining The Factors Interacting with Treatment Success.

**Table 2:** Low-fat and high-quality fat diets in type 2 diabetes mellitus.

Authors (Year) Study Type	Studies/Participants (*N*) Average Duration of Follow-Up (Years)	Population	Diet Type Compared	Findings: T2DM Control (HbA1c/HOMA-IR/FPG/Need for Antidiabetic Drugs/Glycemic Variability)
Brunerova et al. (2007) [70] RCT	1/58 3 months	T2DM and obese non-T2DM adults	High-fat diet enriched with MUFA vs. conventional diet	Decrease in HbA1c from 7.3 ± 0.4% to 6.6 ± 0.3% (*p* < 0.01) on high-fat diet enriched with MUFA vs. from 6.9 ± 0.6% to 6.5 ± 0.5% (*p* > 0.01) on conventional diet
Davis et al. (2009) [79] RTC	1/105 1 year	Overweight adults with T2DM	LCD vs. LFD	There was no significant change in HbA1C in either group.
Brehm et al. (2009) [74] Cohort study	1/1124 1 year	Overweight and obese T2DM adults	High-quality high-MUFA diet vs. HCD	Both diets were equally effective; no significant differences were shown
Iqbal et al. (2010) [80] RTC	1/144 2 years	Obese adults with T2DM	LCD vs. LFD	At month 6, LCD was associated with a clinically significant reduction in HbA1c of −0.5% (compared to −0.1% on LFD), but this was not sustained over time
Itsiopoulos et al. (2011) [68] RCT	1/27 24 weeks	Adults with TDM2.	MED vs. no diet	HbA1c decreased from 7.1% (95% CI: 6.5–7.7) to 6.8% (95% CI: 6.3–7.3) (*p* = 0.012) on MED diet
Guldbrand et al. (2012) [81] Prospective randomized parallel trial	1/61 2 years	Adults with TDM2	LFD vs. LCD	HbA1c LCD at 6 months −4.8 ± 8.3 mmol/mol, *p* = 0.004, at 12 months −2.2 ± 7.7 mmol/mol, *p* = 0.12; LFD at 6 months −0.9 ± 8.8 mmol/mol, *p* = 0.56) Insulin doses were reduced in the LCD group (0 months, LCD 42 ± 65 E, LFD 39 ± 51 E; 6 months, LCD 30 ± 47 E, LFD 38 ± 48 E; *p* = 0.046 for between-group change)
Lasa et al. (2014) [67] Parallel trial	1/177 1 year	Adults free of cardiovascular disease but with T2DM; the participants followed oral anti-diabetic treatments. Participants of the PREDIMED	MED with olive oil vs. MED with nuts vs. LFD	The adiponectin/HOMA-IR (A/HOMA-IR) ratio was significantly increased in the MED with olive oil eatery group and the trend was observed in the MED with nut eatery group (*p* = 0.069) and the LFD group (*p* = 0.061).
Qian et al. (2016) [66] Systematic review and meta-analysis of RCT	24/1460 Up to 3 years	Adults with T2DM	High-MUFA diet vs. HCD	Reductions in fasting plasma glucose: WMD−0,57 mmol/l [95%CI−0.76,−0.39] on High-MUFA diet compared to HCD
Schwingshackl et al. (2017) [77] Network Meta-analysis, Randomized trials	56/4937 3–48 months	Adults with T2DM	LCD vs. LFD	LCD caused significantly greater reduction in HbA_1c_ than LFD: (95% CI) −0.35 (−0.56/−0.14)%
Wang et al. (2018) [78] Prospective, Single-blind randomized controlled trial	1/56 3 months	Chinese T2DM adults	LCD vs. LFD	LCD caused significantly greater reduction in HbA_1c_ than LFD: (95% CI) −0.63% vs. −0.31%, *p* < 0.05.

Abbreviations: BMI—Body Mass Index; FPG—fasting plasma glucose, HbA1c—Glycated hemoglobin; HCD—high-carbohydrate diet; HOMA-IR—Homeostatic Model Assessment for Insulin Resistance; LCD—low-carbohydrate diet; MUFA—monounsaturated fatty acids; MED—Mediterranean diet; PUFA—polyunsaturated fatty acids; T2DM—type 2 diabetes mellitus; RCT—randomized controlled trial.

**Table 3:** Low-fat and high-quality fat diets in cardiovascular disease.

Authors (Year) Study Type	Studies/Participants (*N*) Average Duration of Follow-Up (Years)	Population	Diet Type Compared	Findings			
				CVD Mortality	CHD Risk	Lipids/Blood Pressure	AF
Ascherio et al. (1996) [93] Prospective	1/43757 6 years	Health professional, adults free of diagnosed CVD or T2DM	Diet with high saturated fat vs. diet with low saturated fat intake	-	There was no direct association between reduced saturated fat intake and reduction in CHD risk.	-	-
Pietinen et al. (1997) [98] Prospective	1/21930 6 years	Adult male smokers who were initially free of diagnosed CVD	Low trans fatty acid vs. high trans fatty acid intake	-	RR of coronary death = 1.39 (95% Cl 1.09–1.78) (*p* = 0.004) for the highest vs. lowest quintile of trans fatty acid intake.	-	-
Oh et al. (2005) [84] Observations, prospective epidemiologic studies	1/78778 20 years	US adult women initially free of CVD and T2DM	PUFA vs. trans fat intake	-	PUFA intake was inversely associated with CHD risk (multivariate relative risk (RR) for the highest vs. the lowest quintile = 0.75, 95% CI: 0.60–0.92; *p* trend = 0.004), whereas trans fat intake was associated with an elevated risk of CHD (RR = 1.33, 95% CI: 1.07, −1.66; *p* trend = 0.01).	-	-
Xu et al. (2006) [87] Prospective	1/2938 7 years	Native American adults free of CHD	High saturated fatty acids and MUFA vs. low saturated fatty acids and MUFA intake	Participants aged 47–59 years in the highest quartile of intake of total fat, saturated fatty acids, or MUFA had higher CHD mortality than did those in the lowest quartile (hazard ratio (95% CI): 3.57 (1.21, −10.49), 5.17 (1.64–16.36), and 3.43 (1.17–10.04)).	-	-	-
Leosdottir et al. (2007) [99] Prospective	1/28098 8 years	Middle-aged individuals with no history of CVD	LFD vs. diet with high intake of unsaturated fats	-	There was no association between the risk of CHD and any of the diets.	-	-
Mozaffarian et al., (2010) [96] Meta-analysis, RCT	8/13614 -	Adults who increased PUFA for at least 1 year without major concomitant interventions	PUFA vs. saturated fat intake	-	The overall pooled risk reduction was 19% (RR = 0.81, 95% CI 0.70–0.95, *p* = 0.008), corresponding to 10% reduced CHD risk (RR = 0.90, 95% CI = 0.83–0.97) for each 5% energy of increased PUFA.	-	-
Hooper et al. (2015) [97] Meta-analysis, RCT	15/58509 -	Adults with or without CVD	Saturated fats vs. PUFA intake	Lowering dietary saturated fat reduced the CVD mortality (RR 0.95; 95% CI 0.80 to 1.12).	Lowering dietary saturated fat reduced the CHD risk by 17% (risk ratio (RR) 0.83; 95% confidence interval (CI) 0.72 to 0.96).	-	-
Van Horn et al. (2020) [85] Intervention study “Women’s Health Initiative Dietary Modification”	1/10371 1 year	Adult women without CVD or hypertension	LFD vs. HFD	-	Total fat reduction replaced with increased carbohydrate and some protein, especially plant-based protein, was related to lower CHD risk (in the upper quartile of plant protein intake having a CHD HR of 0.39 (95% CI: 0.22, 0.71), compared with 0.92 (95% CI: 0.57, 1.48) for those in the lower quartile).	-	-

Abbreviations: CHD—Coronary heart disease; CVD—cardiovascular disease; HFD—high-fat diet; LFD—low-fat diet; MUFA—Monounsaturated fatty acids; T2DM—type 2 diabetes mellitus; PUFA—polyunsaturated fatty acids; RCT—randomized controlled trials; US—United States of America.

**Table 4:** Low-carbohydrate diet in obesity.

Authors (Year) Study Type	Studies/Participants (*N*) Average Duration of Follow-Up	Population	Diet Type Compared	Findings (Weight Loss/Hunger)
Samaha et al. (2003) [112] RCT	1/132 6 months	Obese adults with T2DM or metabolic syndrome	LCD vs. LFD	LCD caused significantly greater weight loss than LFD: −5.8 ± 8.6 kg vs. −1.9 ± 4.2 kg; *p* = 0.002.
Yancy et al. (2004) [113] RCT	1/120 24 weeks	Obese adults with lipid disorders and no serious medical condition	LCD vs. LFD	LCD caused significantly greater weight loss than LFD: −12.9% vs. −6.7%; *p* < 0.001.
Brehm et al. (2005) [114] RCT	1/50 4 months	Obese adult women	LCD vs. LFD	LCD caused significantly greater weight loss than LFD: 9.79 ± 0.71 kg vs. 6.14 ± 0.91 kg; *p* < 0.05; and body fat loss: 6.20 ± 0.67 kg vs. 3.23 ± 0.67 kg; *p* < 0.05.
Foster et al. (2010) [121] Randomized parallel-group trial.	1/307 2 years	Obese adults	LCD vs. LFD	There was no statistically significant difference in weight loss.
Bueno et al. (2013) [117] Meta-analysis, RCT	13/1415 At least 12 months	Overweight and obese adults with no restrictions based on sex, race or co-morbidities.	VLCKD vs. LFD	VLCKD caused significantly greater weight loss than LFD: −0.91 (95% CI: −1.65, −0.17) kg.
Gibson et al. (2015) [107] Systematic review, Meta-analysis, Randomized and non-randomized trials	12/967 4–12 weeks	Overweight and obese adults without co-morbidities	VLCKD measured in visual analogue scales. No comparison to other diets	VLCKD caused significant decrease in hunger by 5.5 mm (95% CI: −8.5, −2.5) and desire to eat decreased significantly by 8.9 mm (95% CI: −16.0, −1.8).
Sackner-Bernstein (2015) et al. [115] Meta-analysis, RCT	17/1797 8 weeks to 24 months	Overweight and obese adults with and without co-morbidities.	LCD vs. LFD	LCD caused significantly greater weight loss than LFD: (95% CI) −2.0 (−3.1, −0.9) kg.
Hashimoto et al. (2016) [116] Meta-analysis, RCT	14/1416 2–24 months	Obese adults with or without co-morbidities	LCD vs. control diet	LCD was associated with significantly greater weight loss: (95% CI) −0.7 (−1.07, −0.33) kg; and significantly greater decrease in fat mass: −0.77 (−1.55, −0.32) kg.

Abbreviations: BMI—body mass index; CI—confidence interval; LCD—low-carbohydrate diet; LFD—low-fat diet; RCT—randomized controlled trials; VLCKD—very low-carbohydrate ketogenic diet.

**Table 5:** Low-carbohydrate diet in type 2 diabetes mellitus.

Authors (Year) Study Type	Studies/Participants (*N*) Average Duration of Follow-Up	Population	Diet Type Compared	Findings	
				T2DM Risk Incidence	T2DM Control
Tay et al. (2014) [73] RCT	1/115 2 years	Overweight and obese adults with T2DM	LCD vs. HCD	-	Reduction in the need for antidiabetic drugs: −0.5 ± 0.5 on LCD vs. −0.2 ± 0.5 on HCD; *p ≤* 0.03; reduction in HbA_1c_: −2.6 ± 1.0% (−28.4 ± 10.9 mmol/mol) on LCD vs. −1.9 ± 1.2% (−20.8 ± 13.1 mmol/mol) on HCD; *p* = 0.002.
Nanri (2015) [129] Prospective study	1/64 674 5 years	Japanese adults without previous history of T2DM	LCD vs. other diets	LCD caused decreased risk of T2DM incidence in women (*p* < 0.001).	-
Huntriss et al. (2016) [131] Systematic review, Meta-analysis, RCTs	18/2204 12 weeks to 4 years	Adults with T2DM	LCD vs. other diets	-	LCD caused significantly greater reduction in HbA_1c_ than other diets: (95% CI) −0.28 (−0.53, −0.02)% (*p* = 0.03).
Snorgaard et al. (2017) [134] Systemic review and meta-analysis	10/1376 1 year	Adults with T2DM	LCD vs. HCD	-	LCD caused significantly greater reduction in HbA_1c_ than HCD: 0.34% (3.7 mmol/mol) compared to HCD: (95% CI) 0.06% (0.7 mmol/mol), 0.63% (6.9 mmol/mol).
McArdle et al. (2018) [130] Systematic review, Meta-analysis, RCTs	25/2132 3 months to 4 years	Adults with T2DM	LCD vs VLCD	-	LCD had no effect on HbA_1c_: (95% CI) −0.09 (−0.27, 0.08)% (*p* = 0.30). VLCD lead to significant HbA_1c_ reduction: (95% CI) −0.49 (−0.75, −0.23)% (*p* < 0.001).
Yamada et al. (2018) [132] Systematic review	3/105 6 months	Japanese T2DM adults	LCD vs. calorie-restricted diet	-	LCD caused significantly greater reduction in HbA_1c_ than calorie-restricted diet: 7.0 ± 0.7% vs. 7.5 ± 1.0%; *p* = 0.03.
Turton et al. (2019) [128] Meta-analysis, randomized and non-randomized controlled trials, single-arm intervention studies, retrospective case series analyses, case reports	41/2135 14 days to 24 months	Adults with T2DM	LCD and VLCD, No comparison to other diets	-	All but one of the 41 included LCD interventions were classified as effective in T2DM and none was found to be unsafe.

Abbreviations: CI—confidence interval; HbA_1c_—glycated hemoglobin; HCD—high-carbohydrate diet; LCD—low-carbohydrate diet; RCT—randomized control trials; T2DM—type 2 diabetes mellitus; VLCD — very low-carbohydrate diet.

**Table 6:** Low-carbohydrate diets and cardiovascular risk.

Authors (Year) Study Type	Studies/Participants (*N*) Average Duration of Follow-Up	Population	Diet Type Compared	Findings			
				CVD Mortality	CHD Risk	Lipids/Blood Pressure	AF
Halton et al. (2006) [135] Prospective, Cohort	1/82 802 20 years	Adult female registered nurses from United States	LCD vs. HCD	-	LCD relative risk (95% CI) for CHD: 0.94 (0.76–1.18, *p* = 0.19).	-	-
Lagiou et al. (2012) [140] Prospective, Cohort	1/43 396 15.7 years	Random population sample of Swedish adult women	LCD vs. HCD	-	LCD rate ratio (95% CI) for increased CVD incidence: 1.04 (1–1.08).	-	-
Noto et al. (2013) [138] Systematic review, Meta-analysis, Cohort	4/272 216 At least 1 year	Adults with or without comorbidities	LCD vs. HCD	LCD risk ratio (95% CI) for all-cause mortality: 1.31 (1.07–1.59) LCD risk ratio for CVD mortality and incidence was not statistically significant.	-	-	-
Seidelmann et al. (2018) [137] Prospective, cohort	1/15 428 25 years	Adults from United States who did not report extreme caloric intake	LCD vs. HCD	Mortality risk (95% CI) for LCD: 1.2 (1.09–1.32). Mortality risk (95% CI) for HCD: 1.23 (1.11–1.36).	-	-	-
Zhang et al. (2019) [136] Prospective, cohort	1/13 385 22.4 years	Adults from United States	LCD vs. HCD	-	-	-	Increased risk of AF incident (95% CI) for LCD: HR = 0.82 (0.72-0.94) for AF occurrence related to a 9.4% increase in carbohydrate intake.
Dong et al. (2020) [143] Systematic review, meta-analysis, RCT	12/1 640 at least 3 months	Healthy adults from the USA, Australia, UK, Israel and China.	LCD vs. HCD		-	LCD caused significant changes in: Decrease in triglyceride levels: −0.15 mmol/L (95% CI: −0.23, −0.07); Decrease in systolic blood pressure: −1.41 mmHg (95% CI: —2.26, −0.56); Decrease in diastolic blood pressure: −1.71 mmHg (95% CI: —2.36, −1.06); Increase in plasma HDL-C: 0.1 mmol/L (95% CI: 0.08, 0.12); Decrease in serum total cholesterol: 0.13 mmol/L (95% CI: 0.08, 0.19).	-

Abbreviations: AF—atrial fibrillation; CHD—coronary heart disease; CVD—cardiovascular disease; CI—confidence interval; HCD—high-carbohydrate diet; HDL-C—high-density lipoprotein cholesterol; HR – hazard ratio; LCD—low-carbohydrate diet; RCT—randomized controlled trials.

## References

[1] Bhupathiraju S.N., Hu F.B. (2016). Epidemiology of Obesity and Diabetes and Their Cardiovascular Complications. Circ. Res..

[2] Ward Z.J., Bleich S.N., Cradock A.L., Barrett J.L., Giles C.M., Flax C., Long M.W., Gortmaker S.L. (2019). Projected U.S. State-Level Prevalence of Adult Obesity and Severe Obesity. N. Engl. J. Med..

[3] International Diabetes Federation IDF Diabetes Atlas, 9th edn. Brussels, Belgium: 2019. https://www.diabetesatlas.org.

[4] Micha R., Peñalvo J.L., Cudhea F., Imamura F., Rehm C.D., Mozaffarian D. (2017). Association Between Dietary Factors and Mortality From Heart Disease, Stroke, and Type 2 Diabetes in the United States. JAMA.

[5] World Health Organisation. https://www.who.int/news-room/fact-sheets/detail/cardiovascular-diseases-(cvds).

[6] Wan Y., Tang J., Li J., Li J., Yuan J., Wang F., Li D. (2020). Contribution of diet to gut microbiota and related host cardiometabolic health: Diet-gut interaction in human health. Gut Microbes.

[7] Dalen J.E., Alpert J.S., Goldberg R.J., Weinstein R.S. (2014). The epidemic of the 20(th) century: Coronary heart disease. Am. J. Med..

[8] Konstantinov I.E., Jankovic G.M., Alexander I. (2013). Ignatowski: A pioneer in the study of atherosclerosis. Tex. Heart Inst. J..

[9] Buttar H.S., Li T., Ravi N. (2005). Prevention of cardiovascular diseases: Role of exercise, dietary interventions, obesity and smoking cessation. Exp. Clin. Cardiol..

[10] Sacks F.M., Lichtenstein A.H., Wu J.H.Y., Appel L.J., Creager M.A., Kris-Etherton P.M., Miller M., Rimm E.B., Rudel L.L., Robinson J.G. (2017). Dietary Fats and Cardiovascular Disease: A Presidential Advisory from the American Heart Association. Circulation.

[11] Keys A., Aravanis C., Blackburn H.W., Van Buchem F.S., Buzina R., Djordjević B.D., Dontas A.S., Fidanza F., Karvonen M.J., Kimura N. (1966). Epidemiological studies related to coronary heart disease: Characteristics of men aged 40–59 in seven countries. Acta Medica Scand. Suppl..

[12] Keys A., Anderson J.T., Grande F. (1957). Prediction of serum-cholesterol responses of man to changes in fats in the diet. Lancet.

[13] Shekelle R.B., Shryock A.M., Paul O., Lepper M., Stamler J., Liu S., Raynor W.J. (1981). Diet, serum cholesterol, and death from coronary heart disease. The Western Electric study. N. Engl. J. Med..

[14] Page I.H., Allen E.V., Chamberlain F.L., Keys A., Stamler J., Stare F.J. (1961). Dietary Fat and Its Relation to Heart Attacks and Strokes. Circulation.

[15] Keys A. (1997). Coronary heart disease in seven countries. 1970. Nutrition.

[16] Keys A. (1995). Mediterranean diet and public health: Personal reflections. Am. J. Clin. Nutr.

[17] Salas-Salvadó J., Bulló M., Babio N., Martínez-González M.Á., Ibarrola-Jurado N., Basora J., Estruch R., Covas M.I., Corella D., Arós F. (2011). Reduction in the incidence of type 2 diabetes with the Mediterranean diet: Results of the PREDIMED-Reus nutrition intervention randomized trial. Diabetes Care.

[18] Ferrières J. (2004). The French paradox: Lessons for other countries. Heart.

[19] Renaud S., de Lorgeril M. (1992). Wine, alcohol, platelets, and the French paradox for coronary heart disease. Lancet.

[20] Bhupathiraju S.N., Tucker K.L. (2011). Coronary heart disease prevention: Nutrients, foods, and dietary patterns. Clin. Chim. Acta.

[21] Constant J. (1997). Alcohol, ischemic heart disease, and the French paradox. Clin. Cardiol..

[22] Haseeb S., Alexander B., Baranchuk A. (2017). Wine and Cardiovascular Health: A Comprehensive Review. Circulation.

[23] Haseeb S., Alexander B., Santi R.L., Liprandi A.S., Baranchuk A. (2019). What’s in wine? A clinician’s perspective. Trends Cardiovasc. Med..

[24] La Berge A.F. (2008). How the ideology of low fat conquered america. J. Hist Med. Allied Sci..

[25] Keys A. (1957). Diet and the epidemiology of coronary heart disease. J. Am. Med. Assoc..

[26] Szanto S., Yudkin J. (1969). The effect of dietary sucrose on blood lipids, serum insulin, platelet adhesiveness and body weight in human volunteers. Postgrad. Med. J..

[27] Yudkin J. (1966). Dietetic aspects of atherosclerosis. Angiology.

[28] U.S. Department of Health and Human Services and U.S. Department of Agriculture. 2015–2020 Dietary Guidelines for Americans, 8th ed. December 2015. https://health.gov/our-work/food-and-nutrition/2015-2020-dietary-guidelines/.

[29] McGuire S. (1990). U.S. Department of Agriculture. Nutrition and Your Health: Dietary Guidelines for Americans.

[30] Austin G.L., Ogden L.G., Hill J.O. (2011). Trends in carbohydrate, fat, and protein intakes and association with energy intake in normal-weight, overweight, and obese individuals: 1971–2006. Am. J. Clin. Nutr..

[31] Gross L.S., Li L., Ford E.S., Liu S. (2004). Increased consumption of refined carbohydrates and the epidemic of type 2 diabetes in the United States: An ecologic assessment. Am. J. Clin. Nutr..

[32] Menke A., Casagrande S., Geiss L., Cowie C.C. (2015). Prevalence of and Trends in Diabetes Among Adults in the United States, 1988–2012. JAMA.

[33] Benjamin E.J., Virani S.S., Callaway C.W., Chamberlain A.M., Chang A.R., Cheng S., Chiuve S.E., Cushman M., Delling F.N., Deo R. (2018). Heart Disease and Stroke Statistics-2018 Update: A Report from the American Heart Association. Circulation.

[34] Astrup A., Meinert Larsen T., Harper A. (2004). Atkins and other low-carbohydrate diets: Hoax or an effective tool for weight loss?. Lancet.

[35] Rollo J. (1797). Account of Two Cases of Diabetes Mellitus, with Remarks. Ann. Med. Year.

[36] Kelly T., Unwin D., Finucane F. (2020). Low-Carbohydrate Diets in the Management of Obesity and Type 2 Diabetes: A Review from Clinicians Using the Approach in Practice. Int. J. Environ. Res. Public Health.

[37] Banting W. (1993). Letter on corpulence, addressed to the public. 1869. Obes. Res..

[38] Roberts W.C. (2009). Facts and ideas from anywhere. Bayl. Univ. Med. Cent. Proc..

[39] Evans M. (2018). Keto diets: Good, bad or ugly?. J. Physiol..

[40] Frigolet M.E., Ramos Barragán V.E., Tamez González M. (2011). Low-carbohydrate diets: A matter of love or hate. Ann. Nutr. Metab..

[41] Giugliano D., Maiorino M.I., Bellastella G., Esposito K. (2018). More sugar? No, thank you! The elusive nature of low carbohydrate diets. Endocrine.

[42] Roberts S.B., Das S.K. (2015). One strike against low-carbohydrate diets. Cell Metab..

[43] Walczyk T., Wick J.Y. (2017). The ketogenic diet: Making a comeback. Consult. Pharm..

[44] Ornish D. (2004). Was Dr Atkins right?. J. Am. Diet. Assoc..

[45] Evert A.B., Dennison M., Gardner C.D., Garvey W.T., Lau K.H.K., MacLeod J., Mitri J., Pereira R.F., Rawlings K., Robinson S. (2019). Nutrition Therapy for Adults with Diabetes or Prediabetes: A Consensus Report. Diabetes Care.

[46] Holesh J.E., Aslam S., Martin A. (2020). Physiology, Carbohydrates. StatPearls.

[47] Feinman R.D., Pogozelski W.K., Astrup A., Bernstein R.K., Fine E.J., Westman E.C., Accurso A., Frassetto L., Gower B.A., McFarlane S.I. (2015). Dietary carbohydrate restriction as the first approach in diabetes management: Critical review and evidence base. Nutrition.

[48] World Health Organization (2018). A healthy diet sustainably produced. Int. Arch. Med..

[49] Masood W., Annamaraju P., Uppaluri K.R. (2020). Ketogenic Diet. StatPearls.

[50] Arnett D.K., Blumenthal R.S., Albert M.A., Buroker A.B., Goldberger Z.D., Hahn E.J., Himmelfarb C.D., Khera A., Lloyd-Jones D., McEvoy J.W. (2019). 2019 ACC/AHA Guideline on the Primary Prevention of Cardiovascular Disease: A Report of the American College of Cardiology/American Heart Association Task Force on Clinical Practice Guidelines. J. Am. Coll. Cardiol..

[51] David L.A., Maurice C.F., Carmody R.N., Gootenberg D.B., Button J.E., Wolfe B.E., Ling A.V., Devlin A.S., Varma Y., Fischbach M. (2014). Diet rapidly and reproducibly alters the human gut microbiome. Nature.

[52] Ley R.E., Turnbaugh P.J., Klein S., Gordon J.I. (2006). Microbial ecology: Human gut microbes associated with obesity. Nature.

[53] Qin J., Li Y., Cai Z., Li S., Zhu J., Zhang F., Liang S., Zhang W., Guan Y., Shen D. (2012). A metagenome-wide association study of gut microbiota in type 2 diabetes. Nature.

[54] Tang W.H.W., Kitai T., Hazen S.L. (2017). Gut Microbiota in Cardiovascular Health and Disease. Circ. Res..

[55] Wang Z., Klipfell E., Bennett B.J., Levison B.S., Dugar B., Feldstein A.E., Britt D.B., Fu X., Chung Y.M., Wu Y. (2011). Gut flora metabolism of phosphatidylcholine promotes cardiovascular disease. Nature.

[56] Chistiakov D.A., Bobryshev Y.V., Kozarov E., Sobenin I.A., Orekhov A.N. (2015). Role of gut microbiota in the modulation of atherosclerosis-associated immune response. Front. Microbiol..

[57] Hill J.O., Wyatt H.R., Peters J.C. (2012). Energy balance and obesity. Circulation.

[58] Rolls B.J. (2000). The role of energy density in the overconsumption of fat. J. Nutr.

[59] Quatela A., Callister R., Patterson A., MacDonald-Wicks L. (2016). The Energy Content and Composition of Meals Consumed after an Overnight Fast and Their Effects on Diet Induced Thermogenesis: A Systematic Review, Meta-Analyses and Meta-Regressions. Nutrients.

[60] Murphy E.A., Velazquez K.T., Herbert K.M. (2015). Influence of high-fat diet on gut microbiota: A driving force for chronic disease risk. Curr. Opin. Clin. Nutr. Metab. Care.

[61] Johnston B.C., Kanters S., Bandayrel K., Wu P., Naji F., Siemieniuk R.A., Ball G.D.C., Busse J.W., Thorlund K., Guyatt G. (2014). Comparison of weight loss among named diet programs in overweight and obese adults: A meta-analysis. JAMA J. Am. Med. Assoc..

[62] Gardner C.D., Trepanowski J.F., Del Gobbo L.C., Hauser M.E., Rigdon J., Ioannidis J.P.A., Desai M., King A.C. (2018). Effect of Low-Fat vs Low-Carbohydrate Diet on 12-Month Weight Loss in Overweight Adults and the Association With Genotype Pattern or Insulin Secretion: The DIETFITS Randomized Clinical Trial. JAMA.

[63] Meckling K.A., O’Sullivan C., Saari D. (2004). Comparison of a low-fat diet to a low-carbohydrate diet on weight loss, body composition, and risk factors for diabetes and cardiovascular disease in free-living, overweight men and women. J. Clin. Endocrinol. Metab..

[64] Jensen M.D., Ryan D.H., Apovian C.M., Ard J.D., Comuzzie A.G., Donato K.A., Hu F.B., Hubbard V.S., Jakicic J.M., Kushner R.F. (2014). 2013 AHA/ACC/TOS guideline for the management of overweight and obesity in adults: A report of the American College of Cardiology/American Heart Association Task Force on Practice Guidelines and The Obesity Society. Circulation.

[65] Chawla S., Tessarolo Silva F., Amaral Medeiros S., Mekary R.A., Radenkovic D. (2020). The Effect of Low-Fat and Low-Carbohydrate Diets on Weight Loss and Lipid Levels: A Systematic Review and Meta-Analysis. Nutrients.

[66] Qian F., Korat A.A., Malik V., Hu F.B. (2016). Metabolic Effects of Monounsaturated Fatty Acid-Enriched Diets Compared With Carbohydrate or Polyunsaturated Fatty Acid-Enriched Diets in Patients With Type 2 Diabetes: A Systematic Review and Meta-analysis of Randomized Controlled Trials. Diabetes Care.

[67] Lasa A., Miranda J., Bulló M., Casas R., Salas-Salvado J., Larretxi I., Estruch R., Ruiz-Gutierrez V., Portillo M.P. (2014). Comparative effect of two Mediterranean diets versus a low-fat diet on glycaemic control in individuals with type 2 diabetes. Eur. J. Clin. Nutr..

[68] Itsiopoulos C., Brazionis L., Kaimakamis M., Cameron M., Best J.D., O’Dea K., Rowley K. (2011). Can the Mediterranean diet lower HbA1c in type 2 diabetes? Results from a randomized cross-over study. Nutr. Metab. Cardiovasc. Dis..

[69] Pi-Sunyer X., Blackburn G., Brancati F.L., Bray G.A., Bright R., Clark J.M., Burtis J.M., Espeland M.A., Foreyt Y.P., Look AHEAD Research Group (2007). Reduction in weight and cardiovascular disease risk factors in individuals with type 2 diabetes: One-year results of the look AHEAD trial. Diabetes Care.

[70] Brunerova L., Smejkalova V., Potockova J., Andel M. (2007). A comparison of the influence of a high-fat diet enriched in monounsaturated fatty acids and conventional diet on weight loss and metabolic parameters in obese non-diabetic and Type 2 diabetic patients. Diabet. Med..

[71] Kodama S., Saito K., Tanaka S., Maki M., Yachi Y., Sato M., Sugawara A., Totsuka K., Shimano H., Ohashi Y. (2009). Influence of fat and carbohydrate proportions on the metabolic profile in patients with type 2 diabetes: A meta-analysis. Diabetes Care.

[72] Wheeler M.L., Dunbar S.A., Jaacks L.M., Karmally W., Mayer-Davis E.J., Wylie-Rosett J., Yancy W.S. (2012). Macronutrients, food groups, and eating patterns in the management of diabetes: A systematic review of the literature, 2010. Diabetes Care.

[73] Tay J., Luscombe-Marsh N.D., Thompson C.H., Noakes M., Buckley J.D., Wittert G.A., Yancy W.S., Brinkworth G.D. (2014). A very low-carbohydrate, low-saturated fat diet for type 2 diabetes management: A randomized trial. Diabetes Care.

[74] Brehm B.J., Lattin B.L., Summer S.S., Boback J.A., Gilchrist G.M., Jandacek R.J., D’Alessio D.A. (2009). One-year comparison of a high-monounsaturated fat diet with a high-carbohydrate diet in type 2 diabetes. Diabetes Care.

[75] Katan M.B., Zock P.L., Mensink R.P. (1995). Dietary oils, serum lipoproteins, and coronary heart disease. Am. J. Clin. Nutr..

[76] Hu F.B., Stampfer M.J., Manson J.E., Rimm E., Colditz G.A., Rosner B.A., Hennekens C.H., Willett W.C. (1997). Dietary fat intake and the risk of coronary heart disease in women. N. Engl. Med..

[77] Schwingshackl L., Chaimani A., Hoffmann G., Schwedhelm C., Boeing H. (2018). A network meta-analysis on the comparative efficacy of different dietary approaches on glycaemic control in patients with type 2 diabetes mellitus. Eur. J. Epidemiol..

[78] Wang L.L., Wang Q., Hong Y., Ojo O., Jiang Q., Hou Y.Y., Huang Y.H., Wang X.H. (2018). The effect of low-carbohydrate diet on glycemic control in patients with type 2 diabetes mellitus. Nutrients.

[79] Davis N.J., Tomuta N., Schechter C., Isasi C.R., Segal-Issaacson C.J., Stein D., Zonszein J., Wylie-Rosett J. (2009). Comparative study of the effects of a 1-year dietary intervention of a low-carbohydrate diet versus a low-fat diet on weight and glycemic control in type 2 diabetes. Diabetes Care.

[80] Iqbal N., Vetter M.L., Moore R.H., Chittams J.L., Dalton-Bakes C.V., Dowd M., Williams-Smith C., Cardillo S., Wadden T.A. (2010). Effects of a low-intensity intervention that prescribed a low-carbohydrate vs. a low-fat diet in obese, diabetic participants. Obesity.

[81] Guldbrand H., Dizdar B., Bunjaku B., Lindström T., Bachrach- Lindström M., Fredrikson M., Ostgren C.J., Nystrom F.H. (2012). In type 2 diabetes, randomisation to advice to follow a low-carbohydrate diet transiently improves glycaemic control compared with advice to follow a low-fat diet producing a similar weight loss. Diabetologia.

[82] Shan Z., Guo Y., Hu F.B., Liu L., Qi Q. (2020). Association of Low-Carbohydrate and Low-Fat Diets with Mortality Among US Adults. JAMA Intern. Med..

[83] Jakobsen M.U., O’Reilly E.J., Heitmann B.L., Pereira M.A., Bälter K., Fraser G.E., Goldbourt U., Hallmans G., Knekt P., Liu S. (2009). Major types of dietary fat and risk of coronary heart disease: A pooled analysis of 11 cohort studies. Am. J. Clin. Nutr..

[84] Oh K., Hu F.B., Manson J.E., Stampfer M.J., Willett W.C. (2005). Dietary fat intake and risk of coronary heart disease in women: 20 years of follow-up of the nurses’ health study. Am. J. Epidemiol..

[85] Van Horn L., Aragaki A.K., Howard B.V., Allison M.A., Isasi C.R., Manson J.E., Neuhouser M.L., Mossavar-Rahmani Y., Thomson C.A., Vitolin M.Z. (2020). Eating Pattern Response to a Low-Fat Diet Intervention and Cardiovascular Outcomes in Normotensive Women: The Women’s Health Initiative. Curr. Dev. Nutr..

[86] Shan Z., Rehm C.D., Rogers G., Ruan M., Wang D.D., Hu F.B., Mozaffarian D., Zhang F.F., Bhupathiraju S.N. (2019). Trends in Dietary Carbohydrate, Protein, and Fat Intake and Diet Quality Among US Adults, 1999–2016. JAMA.

[87] Xu J., Eilat-Adar S., Loria C., Goldbourt U., Howard B.V., Fabsitz R.R., Zephier E.M., Mattil C., Lee E.T. (2006). Dietary fat intake and risk of coronary heart disease: The Strong Heart Study. Am. J. Clin. Nutr..

[88] Leren P. (1970). The Oslo diet-heart study. Eleven-year report. Circulation.

[89] Siri-Tarino P.W., Sun Q., Hu F.B., Krauss R.M. (2010). Meta-analysis of prospective cohort studies evaluating the association of saturated fat with cardiovascular disease. Am. J. Clin. Nutr..

[90] Hamley S. (2017). The effect of replacing saturated fat with mostly n-6 polyunsaturated fat on coronary heart disease: A meta-analysis of randomised controlled trials. Nutr. J..

[91] Kromhout D., Bosschieter E.B., de Lezenne Coulander C. (1985). The inverse relation between fish consumption and 20-year mortality from coronary heart disease. N. Engl. J. Med..

[92] Casas R., Castro-Barquero S., Estruch R., Sacanella E. (2018). Nutrition and Cardiovascular Health. Int. J. Mol. Sci..

[93] Ascherio A., Rimm E.B., Giovannucci E.L., Spiegelman D., Stampfer M., Willett W.C. (1996). Dietary fat and risk of coronary heart disease in men: Cohort follow up study in the United States. BMJ.

[94] Burke M.F., Burke F.M., Soffer D.E. (2017). Review of Cardiometabolic Effects of Prescription Omega-3 Fatty Acids. Curr. Atheroscler. Rep..

[95] Spigoni V., Lombardi C., Cito M., Picconi A. (2014). N-3 PUFA increase bioavailability and function of endothelial progenitor cells. Food Funct..

[96] Mozaffarian D., Micha R., Wallace S. (2010). Effects on coronary heart disease of increasing polyunsaturated fat in place of saturated fat: A systematic review and meta-analysis of randomized controlled trials. PLoS Med..

[97] Hooper L., Martin N., Abdelhamid A., Davey Smith G. (2015). Reduction in saturated fat intake for cardiovascular disease. Cochrane Database Syst. Rev..

[98] Pietinen P., Ascherio A., Korhonen P., Hartman A.M., Willett W.C., Albanes D., Virtamo J. (1997). Intake of fatty acids and risk of coronary heart disease in a cohort of Finnish men. The Alpha-Tocopherol, Beta-Carotene Cancer Prevention Study. Am. J. Epidemiol..

[99] Leosdottir M., Nilsson P.M., Nilsson J.A., Berglund G. (2007). Cardiovascular event risk in relation to dietary fat intake in middle-aged individuals: Data from The Malmö Diet and Cancer Study. Eur. J. Cardiovasc. Prev. Rehabil..

[100] Estruch R., Ros E., Salas-Salvadó J., Covas M.I., Corella D., Arós F., Gómez-Gracia E., Ruiz-Gutiérrez V., Fiol M., Lapetra J. (2013). Primary Prevention of Cardiovascular Disease with a Mediterranean Diet. N. Engl. Med..

[101] Estruch R., Ros E., Salas-Salvadó J., Covas M.I., Corella D., Arós F., Gómez-Gracia E., Ruiz-Gutiérrez V., Fiol M., Lapetra J. (2018). Retraction and Republication: Primary Prevention of Cardiovascular Disease with a Mediterranean Diet. N. Engl. J. Med..

[102] Adam-Perrot A., Clifton P., Brouns F. (2006). Low-carbohydrate diets: Nutritional and physiological aspects. Obes. Rev..

[103] Westman E.C., Feinman R.D., Mavropoulos J.C., Vernon M.C., Volek J.S., Wortman J.A., Yancy W.S., Phinney S.D. (2007). Low-carbohydrate nutrition and metabolism. Am. J. Clin. Nutr..

[104] Paoli A., Rubini A., Volek J.S., Grimaldi K.A. (2013). Beyond weight loss: A review of the therapeutic uses of very-low-carbohydrate (ketogenic) diets. Eur. J. Clin. Nutr..

[105] Ebbeling C.B., Feldman H.A., Klein G.L., Wong J., Bielak L., Steltz S.K., Luoto P.K., Wolfe R.R., Wong W.W., Ludwig D.S. (2018). Effects of a low carbohydrate diet on energy expenditure during weight loss maintenance: Randomized trial. BMJ.

[106] Oh R., Gilani B., Uppaluri K.R. (2020). Low Carbohydrate Diet. StatPearls.

[107] Gibson A.A., Seimon R.V., Lee C.M.Y., Ayre J., Franklin J., Markovic T.P., Caterson I.D., Sainsbury A. (2015). Do ketogenic diets really suppress appetite? A systematic review and meta-analysis. Obes. Rev..

[108] Leidy H.J., Clifton P.M., Astrup A., Wycherley T.P., Westerterp-Plantenga M.S., Luscombe-Marsh N.D., Woods S.C., Mattes R.D. (2015). The role of protein in weight loss and maintenance. Am. J. Clin. Nutr..

[109] Ludwig D.S. (2002). The glycemic index: Physiological mechanisms relating to obesity, diabetes, and cardiovascular disease. JAMA.

[110] Seid H., Rosenbaum M. (2019). Low carbohydrate and low-fat diets: What we don’t know and why we should know it. Nutrients.

[111] Brehm B.J., Seeley R.J., Daniels S.R., D’Alessio D.A. (2003). A randomized trial comparing a very low carbohydrate diet and a calorie-restricted low fat diet on body weight and cardiovascular risk factors in healthy women. J. Clin. Endocrinol. Metab..

[112] Samaha F.F., Iqbal N., Seshadri P., Chicano K.L., Daily D.A., McGrory J., Williams T., Williams M., Gracely E.J., Stern L. (2003). A low-carbohydrate as compared with a low-fat diet in severe obesity. N. Engl. J. Med..

[113] Yancy W.S., Olsen M.K., Guyton J.R., Bakst R.P., Westman E.C. (2004). A low-carbohydrate, ketogenic diet versus a low-fat diet to treat obesity and hyperlipidemia: A randomized, controlled trial. Ann. Intern. Med..

[114] Brehm B.J., Spang S.E., Lattin B.L., Seeley R.J., Daniels S.R., D’Alessio D.A. (2005). The role of energy expenditure in the differential weight loss in obese women on low-fat and low-carbohydrate diets. J. Clin. Endocrinol. Metab..

[115] Sackner-Bernstein J., Kanter D., Kaul S. (2015). Dietary intervention for overweight and obese adults: Comparison of low- carbohydrate and low-fat diets. a meta- analysis. PLoS ONE.

[116] Hashimoto Y., Fukuda T., Oyabu C., Tanaka M., Asano M., Yamazaki M., Fukui M. (2016). Impact of low-carbohydrate diet on body composition: Meta-analysis of randomized controlled studies. Obes. Rev..

[117] Bueno N.B., De Melo I.S.V., De Oliveira S.L., Da Rocha Ataide T. (2013). Very-low-carbohydrate ketogenic diet v. low-fat diet for long-term weight loss: A meta-analysis of Randomised controlled trials. Br. J. Nutr..

[118] Hession M., Rolland C., Kulkarni U., Wise A., Broom J. (2009). Systematic review of randomized controlled trials of low-carbohydrate vs. low-fat/low-calorie diets in the management of obesity and its comorbidities. Obes. Rev..

[119] Tobias D.K., Chen M., Manson J.A.E., Ludwig D.S., Willett W., Hu F.B. (2015). Effect of low-fat diet interventions versus other diet interventions on long-term weight change in adults: A systematic review and meta-analysis. Lancet Diabetes Endocrinol..

[120] Burgess B., Raynor H.A., Tepper B.J. (2017). PROP Nontaster Women Lose More Weight Following a Low-Carbohydrate Versus a Low-Fat Diet in a Randomized Controlled Trial. Obesity.

[121] Foster G.D., Wyatt H.R., Hill J.O., Makris A.P., Rosenbaum D.L., Brill C., Stein R.I., Mohammed B.S., Miller B., Rader D.J. (2010). Weight and metabolic outcomes after 2 years on a low-carbohydrate versus low-fat diet: A randomized trial. Ann. Intern. Med..

[122] Kirkpatrick C.F., Bolick J.P., Kris-Etherton P.M., Sikand G., Aspry K.E., Soffer D.E., Willard K.E., Maki K.C. (2019). Review of current evidence and clinical recommendations on the effects of low-carbohydrate and very-low-carbohydrate (including ketogenic) diets for the management of body weight and other cardiometabolic risk factors: A scientific statement from the Nat. J. Clin. Lipidol..

[123] Cosentino F., Grant P.J., Aboyans V., Bailey C.J., Ceriello A., Delgado V., Federici M., Filippatos G., Grobbee D.E., Hansen T.B. (2020). 2019 ESC Guidelines on diabetes, pre-diabetes, and cardiovascular diseases developed in collaboration with the EASD. Eur. Heart J..

[124] White J.R. (2014). A brief history of the development of diabetes medications. Diabetes Spectr..

[125] Westman E.C., Yancy W.S., Humphreys M. (2006). Dietary treatment of diabetes mellitus in the pre-insulin era (1914–1922). Perspect. Biol. Med..

[126] Allen F.M. (1914). Studies concerning diabetes. J. Am. Med. Assoc..

[127] Joslin E.P. (1916). The Treatment of Diabetes Mellitus. Can. Med. Assoc. J..

[128] Turton J., Brinkworth G.D., Field R., Parker H., Rooney K. (2019). An evidence-based approach to developing low-carbohydrate diets for type 2 diabetes management: A systematic review of interventions and methods. Diabetes Obes. Metab..

[129] Nanri A., Mizoue T., Kurotani K., Goto A., Oba S., Noda M., Sawada N., Tsugane S., Song Y. (2015). Low-carbohydrate diet and type 2 diabetes risk in Japanese men and women: The Japan Public Health Center-Based Prospective Study. PLoS ONE.

[130] McArdle P.D., Greenfield S.M., Rilstone S.K., Narendran P., Haque M.S., Gill P.S. (2019). Carbohydrate restriction for glycaemic control in Type 2 diabetes: A systematic review and meta-analysis. Diabet. Med..

[131] Huntriss R., Campbell M., Bedwell C. (2018). The interpretation and effect of a low-carbohydrate diet in the management of type 2 diabetes: A systematic review and meta-analysis of randomised controlled trials. Eur. J. Clin. Nutr..

[132] Yamada S., Kabeya Y., Noto H. (2018). Dietary approaches for Japanese patients with diabetes: A systematic review. Nutrients.

[133] Westman E.C., Tondt J., Maguire E., Yancy W.S. (2018). Implementing a low-carbohydrate, ketogenic diet to manage type 2 diabetes mellitus. Expert Rev. Endocrinol. Metab..

[134] Snorgaard O., Poulsen G.M., Andersen H.K., Astrup A. (2017). Systematic review and meta-analysis of dietary carbohydrate restriction in patients with type 2 diabetes. BMJ Open Diabetes Res. Care.

[135] Halton T.L., Willett W.C., Liu S., Manson J.E., Albert C.M., Rexrode K., Hu F.B. (2006). Low-Carbohydrate-Diet Score and the Risk of Coronary Heart Disease in Women. N. Engl. J. Med..

[136] Zhang S., Zhuang X., Lin X., Zhong X., Zhou H., Sun X., Xiong Z., Huang Y., Fan Y., Guo Y. (2019). Low-Carbohydrate Diets and Risk of Incident Atrial Fibrillation: A Prospective Cohort Study. J. Am. Heart Assoc..

[137] Seidelmann S.B., Claggett B., Cheng S., Henglin M., Shah A., Steffen M.L., Folsom A.R., Rimm E.B., Willett W.C., Solomon S.D. (2018). Dietary carbohydrate intake and mortality: A prospective cohort study and meta-analysis. Lancet Public Heal..

[138] Noto H., Goto A., Tsujimoto T., Noda M. (2013). Low-Carbohydrate Diets and All-Cause Mortality: A Systematic Review and Meta-Analysis of Observational Studies. PLoS ONE.

[139] Hu T., Bazzano L.A. (2014). The low-carbohydrate diet and cardiovascular risk factors: Evidence from epidemiologic studies. Nutr. Metab. Cardiovasc. Dis..

[140] Lagiou P., Sandin S., Lof M., Trichopoulos D., Adami H.O., Weiderpass E. (2012). Low carbohydrate-high protein diet and incidence of cardiovascular diseases in Swedish women: Prospective cohort study. BMJ.

[141] Retterstøl K., Svendsen M., Narverud I., Holven K.B. (2018). Effect of low carbohydrate high fat diet on LDL cholesterol and gene expression in normal-weight, young adults: A randomized controlled study. Atherosclerosis.

[142] Durrer C., Lewis N., Wan Z., Ainslie P.N., Jenkins N.T., Little J.P. (2019). Short-term low-carbohydrate high-fat diet in healthy young males renders the endothelium susceptible to hyperglycemia-induced damage, an exploratory analysis. Nutrients.

[143] Dong T., Guo M., Zhang P., Sun G., Chen B. (2020). The effects of low-carbohydrate diets on cardiovascular risk factors: A meta-analysis. PLoS ONE.

[144] Meng Y., Bai H., Wang S., Li Z., Wang Q., Chen L. (2017). Efficacy of low carbohydrate diet for type 2 diabetes mellitus management: A systematic review and meta-analysis of randomized controlled trials. Diabetes Res. Clin. Pract..

[145] Lorber D. (2014). Importance of cardiovascular disease risk management in patients with type 2 diabetes mellitus. Diabetes Metab. Syndr. Obes. Targets Ther..

[146] Unwin D.J., Tobin S.D., Murray S.W., Delon C., Brady A.J. (2019). Substantial and sustained improvements in blood pressure, weight and lipid profiles from a carbohydrate restricted diet: An observational study of insulin resistant patients in primary care. Int. J. Environ. Res. Public Health.

[147] Hamdy O., Tasabehji M.W., Elseaidy T., Tomah S., Ashrafzadeh S., Mottalib A. (2018). Fat Versus Carbohydrate-Based Energy-Restricted Diets for Weight Loss in Patients with Type 2 Diabetes. Curr. Diab. Rep..

[148] Dehghan M., Mente A., Zhang X., Swaminathan S., Li W., Mohan V., Iqbal R., Kumar R., Wentzel- Viljoen E., Rosengren A. (2017). Associations of fats and carbohydrate intake with cardiovascular disease and mortality in 18 countries from five continents (PURE): A prospective cohort study. Lancet.

[149] Ho F.K., Gray S.R., Welsh P., Petermann- Rocha F., Foster H., Waddell H., Anderson J., Lyall D., Sattar N., Gill J.M.R. (2020). Associations of fat and carbohydrate intake with cardiovascular disease and mortality: Prospective cohort study of UK Biobank participants. BMJ.

[150] Sieri S., Agnoli C., Grioni S., Weiderpass E., Mattiello A., Sluijs I., Sanchez M.J., Jakobsen M.U., Sweeting M., van der Schouw Y.T. (2020). Glycemic index, glycemic load, and risk of coronary heart disease: A pan-European cohort study. Am. J. Clin. Nutr..

[151] Srour B., Fezeu L.K., Kesse-Guyot E., Allès B., Méjean C., Andrianasolo M.R., Chazelas E., Deschasaux M., Hercberg S., Galan P. (2019). Ultra-processed food intake and risk of cardiovascular disease: Prospective cohort study (NutriNet-Santé). BMJ.

[152] Riva A., Borgo F., Lassandro C., Verduci E., Morace G., Borghi E., Berry D. (2017). Pediatric obesity is associated with an altered gut microbiota and discordant shifts in Firmicutes populations. Environ. Microbiol..

[153] Karlsson F.H., Tremaroli V., Nookaew I., Bergström G., Behre C.J., Fagerberg B., Nielsen J., Bäckhed F. (2013). Gut metagenome in European women with normal, impaired and diabetic glucose control. Nature.

[154] Larsen N., Vogensen F.K., van den Berg F.W., Nielsen D.S., Andreasen A.S., Pedersen B.K., Al- Soud W.A., Sørensen S.J., Hansen L.H., Jakobsen M. (2010). Gut microbiota in human adults with type 2 diabetes differs from non-diabetic adults. PLoS ONE.

[155] Lin H.V., Frassetto A., Kowalik E.J.J., Nawrocki A.R., Lu M.M., Kosinski J.R., Hubert J.A., Szeto D., Yao X., Forrest G. (2012). Butyrate and propionate protect against diet-induced obesity and regulate gut hormones via free fatty acid receptor 3-independent mechanisms. PLoS ONE.

[156] Alhassan S., Kim S., Bersamin A., King A.C., Gardner C.D. (2008). Dietary adherence and weight loss success among overweight women: Results from the A to Z weight loss study. Int. J. Obes..

[157] Grembi J.A., Nguyen L.H., Haggerty T.D., Gardner C.D., Holmes S.P., Parsonnet J. (2020). Gut microbiota plasticity is correlated with sustained weight loss on a low-carb or low-fat dietary intervention. Sci. Rep..

[158] Wan Y., Wang F., Yuan J., Lie J., Jiang D., Zhang J., Li H., Wang R., Tang J., Huang T. (2019). Effects of dietary fat on gut microbiota and faecal metabolites, and their relationship with cardiometabolic risk factors: A 6-month randomised controlled-feeding trial. Gut.

[159] Sonnenburg E.D., Smits S.A., Tikhonov M., Higginbottom S.K., Wingreen N.S., Sonnenburg J.L. (2016). Diet-induced extinctions in the gut microbiota compound over generations. Nature.

[160] Kazemian N., Mahmoudi M., Halperin F., Wu J.C., Pakpour S. (2020). Gut microbiota and cardiovascular disease: Opportunities and challenges. Microbiome.

[161] Koeth R.A., Wang Z., Levison B.S., Buffa J.A., Org E., Sheehy B.T., Britt E.B., Fu X., Wu Y., Li L. (2013). Intestinal microbiota metabolism of L-carnitine, a nutrient in red meat, promotes atherosclerosis. Nat. Med..

[162] Schiattarella G.G., Sannino A., Toscano E., Giugliano G., Gargiulo G., Franzone A., Trimarco B., Esposito G., Perrino C. (2017). Gut microbe-generated metabolite trimethylamine-N-oxide as cardiovascular risk biomarker: A systematic review and dose-response meta-analysis. Eur. Heart J..

[163] Laudisi F., Stolfi C., Monteleone G. (2019). Impact of Food Additives on Gut Homeostasis. Nutrients.

[164] Zinöcker M.K., Lindseth I.A. (2018). The western diet–microbiome-host interaction and its role in metabolic disease. Nutrients.

